# Efficacy of plasma rich in growth factor used for dry socket management: a systematic review

**DOI:** 10.4317/medoral.23015

**Published:** 2019-10-27

**Authors:** Ji Liang Xu, Rong Xia

**Affiliations:** 1 Department of Stomatology, the Second Hospital of Anhui Medical University, 678 Furong Road, Hefei 230601, People’s Republic of China; 2 Department of Stomatology, Lianhua community health service centre of the Second Hospital of Anhui Medical University, 736 lianhua Road, Hefei 230601, People’s Republic of China

## Abstract

**Background:**

The main aim of this systematic review was to assess the dry socket management using plasma rich in growth factor (PRGF) in terms of pain relief, alveolar fossa healing, inflammation, the incidence of dry socket.

**Material and Methods:**

PubMed, Cochrane Library, Elsevier Science Direct, China Biology Medicine (CBM), China National Knowledge Infrastructure (CNKI) and VIP database were searched for the related articles without language limitation. Two reviewers independently searched and evaluated relevant studies. This review has been registered in the website PROSPERO (CRD42018087252).

**Results:**

28 articles were retrieved on PubMed and 98 on other electronic databases in the initial search. In the end, 4 randomized controlled trials (RCTs) were included, with a total of 139 patients enrolled. The descriptive results indicated that the use of PRGF may help reduce pain and inflammation after tooth extraction. To some extent, it is beneficial to the management of dry socket after extraction.

**Conclusions:**

Quality assessment indicated all the included studies were judged to be at high risk of bias with low quality. Hence, it was impossible to make a recommendation for clinical use of PRGF based on the current evidence. Clearly, a multicenter clinical randomized controlled trial is needed urgent to evaluate the safety and efficacy of PRGF for dry socket management.

** Key words:**plasma rich in growth factor, PRGF, dry socket, systematic review.

## Introduction

The unscientific term “dry socket” also called alveolar osteitis, was first proposed by Crawford in 1896. Dry socket is characterized by an extraction alveolus lack of a blood clot and along with sudden intense, lancing and radiated pain after 2-3 days following tooth extraction in and around the extraction sites, accompanying strong smell of corruption (1-4). The most common findings in clinical examinations are the extraction sockets without any blood clots and sometimes part blood clots with spoilage and necrosis in the extraction sockets (5). On account of the different diagnostic criteria used, the incidence of dry socket reported in the literature is inconsistent. It is reported that the incidence of dry socket is about 20%-35% after impacted mandibular third molars extraction (2,6,7), whereas its occurrence for all extraction is from 3% to 5% (8,9).

Patients infected with dry socket after extraction often seek urgent treatment for severe pain, halitosis and dysgeusia (bad taste) (4). To date, various modalities were proposed for dry socket management. These treatments included topical using pain reducing dressing such as zinc oxide eugenol (ZOE) dressing (10-13) and chitosan dressing (14), topical or systemic antibiotics agents, and wound healing promoting drugs for example exogenous tretinoin acid (15), honey (16,17),herbal exacts (18). Surgical debridement treatment for dry socket, refers to some or all of the following procedures: block anesthesia for the socket, debridement, irrigation and placing a dressing with or without sutures (19).

Autologous platelet concentrates (PCs) are being widely used in the field of dental and tissue regenerative medicine (20-22). PRGF is a leukocyte-free, of standard composition and dosage, 100% autologous platelet-rich plasma (PRP) (23-25). However, PRGF is regarded as a secure and optimized product that avoids many of the limitations of using other PRPs (26). It has been reported that PRGF has been displayed tissue regeneration in implant placement and the maxillary sinus membrane damage repair (27-31) and antibacterial activity (32,33) in a series of post-operations in oral surgery.

There were conflicting results for the efficacy of PRGF used for dry socket management following the third molars extraction (12,26,34,35), due to small sample size, short follow-up and inconsistent outcome variables.

The main aim of this systematic review was to assess the dry socket management using PRGF in terms of pain relief, alveolar fossa healing, inflammation, the incidence of dry socket.

## Material and Methods

This systematic review was performed in agreement with PRISMA statement guidelines (36). The protocol and methods used were registered in website PROSPERO (CRD42018087252), which is an international prospective register of systematic reviews.

- Search methods and key words

Systematic and comprehensive retrieval was carried out in the following electronic database: PubMed, Cochrane Library, Elsevier Science Direct, China Biology Medicine (CBM), China National Knowledge Infrastructure (CNKI) and VIP database (Chinese scientific journal database). The search strategy on PubMed is as follows: (dry socket OR alveolar osteitis OR fibrinolytic alveolitis OR alveolitis sicca dolorosa OR localized osteomyelitis OR delayed extraction wound healing OR localized osteitis alveoli OR septic socket OR necrotizing socket OR tooth extraction* OR teeth extraction* OR dry socket [Mesh]) AND (plasma rich in growth factor* OR plasma rich growth factor* OR PRGF). In other electronic databases, the mesh terms search was not used.

In addition, a manual search was performed to find related articles in the following publications: British Journal of Oral and Maxillofacial Surgery, International Journal of Oral and Maxillofacial Surgery, Journal of oral and maxillofacial surgery, Oral Surgery, Oral Medicine, Oral Pathology and Oral Radiology, European Journal of Dentistry, Journal of Dental Research, Journal of the American Dental Association, Australian Dental Journal, Oral and Maxillofacial Surgery, British Dental Journal, Oral Diseases, Indian Journal of Dental Research.

Besides, a manual search and screening of the references reported in the studies identified was also complemented. The above retrieval process was performed by two reviewers independently (XJL and LJT). An upper date limit of 26 March 2019 was applied, with no lower date limit. There was no language restriction.

Inclusion and exclusion criteria

Studies with the following characteristics were included: 1-Population: studies of humans that included adult patients who had undergone extractions of one or more teeth; 2-Intervention: application of PRGF to post extraction sockets; 3-Comparison: application of a placebo or other treatments to post extraction sockets; 4-Outcome: pain relief, alveolar fossa healing, inflammation, the incidence of dry socket; 5-Study design: only randomized clinical trials (RCTs) were included, with the other factors matched between the groups.

Exclusion criteria: 1-reviews, editorials, case reports, letters and conference abstracts; 2-animals studies were excluded; 3-studies without control group were excluded.

- Data extraction 

The data from all included studies were extracted independently by two reviews (XJL and LJT) using predefined data extraction form. The following information was extracted from each study (when available): first author, publication year, location, design method, characteristics of population, smoking status, oral contraceptive administration, extraction sites, intervention characteristics, follow-up period, outcome measures.

- Quality assessment

The methodological quality of all selected studies were evaluated independently by two reviewers (XJL and LJT), and these were cross-checked. Disagreements between the two reviewers were resolved by consensus with a third reviewer (XR). Quality assessment tool for randomized controlled trials was on the basis of the risk of bias assessment scale recommended in the Cochrane handbook for systematic reviews. The classification of the risk of bias potential was based on the following criteria: sequence generation, allocation concealment, blinding of participants and personnel, blinding of outcome assessment, incomplete outcome data addressed, selective reporting, and other sources of bias. If all assessment items are reported as ‘yes’, the study is judged to be at low risk of bias. If one or more items are reported as ‘unclear’, the study is considered to be at moderate risk of bias. If one or more items are reported as ‘no’, the study is regarded as being at high risk of bias.

## Results

- Study selection

**Figure 1:**
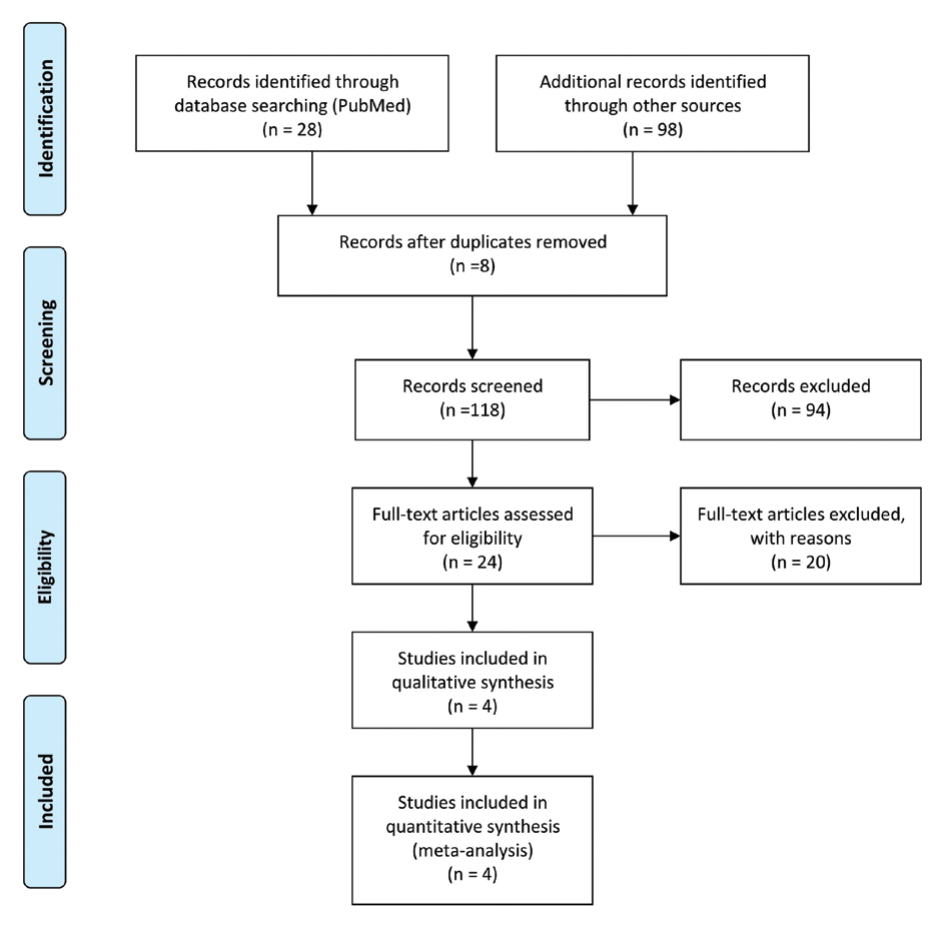
PRISMA flow chart of the search strategy.

126 titles were retrieved in the initial search. All the titles were poured into the professional document management NoteExpress (a Chinese software similar to EndNote, *http://lib.ahmu.edu.cn/*). Its duplicate checking function excluded 8 titles. After duplicate removal, 118 titles were screened by title and abstract, leading to 94 titles excluded. Moreover, 24 potentially eligible titles were retrieved through the consulting of reference lists of included studies. Finally, only 4 articles were included for the qualitative synthesis (Fig. 1).

- Characteristics of all the included studies

Basic details of all the selected studies were shown in Table 1. All the four studies were RCTs, two of which adopted split-mouth design. A total of 139 patients were enrolled in the four studies. The duration of the study was 8 years, from 2010 to 2018. Two studies were carried out in Europe (UK, Italy), one in India and the other in Iran. Two studies included smokers, but did not detail the smoking status. Only one study included women taking oral contraceptives. Not every article provided information about the number of sockets and the extraction sites. The follow-up periods varied among the four articles, with the shortest of one day and the longest of 15 days. All clinical outcome measures in four studies comprised pain relief and healing situation.

- Quality assessment

Every study was evaluated for potential risk of bias. The results of the quality assessment of all the four studies was presented in Table 2. Unfortunately, all included studies were judged to be at high risk of bias with low quality. Kappa statistic value for the inter-reviewer agreement was 0.851, which indicates “almost perfect” based on Landis and Koch scale (37).

- Quantitative synthesis

The results of studies included for analysis were presented in Table 3 and Table 3 cont. This systematic review did an attempt to conduct a meta-analysis by virtue of STATA 12.0 software, which could summarize the extracted data from the included studies, enhancing statistical power. However, based on the above fact that too small sample, low-quality literature and high heterogeneity among the four studies, a meta-analysis was considered to be inappropriate here.

- Qualitative description 

Pain relief

VAS scores system of 10 cm was used for evaluation of pain level in all included studies. Mozzati *et al*. found that the pain level was lower for PRGF sites than for control sites at all times examined (1d to 7d), however, this difference was statistically significant only at 7d with *p* value of <0.05. Haraji *et al*. reported that the intensity of post-extraction pain in PRGF group was significantly less than that in control group with *p* value of < 0.00 at each post-extraction day (2d, 3d, 4d). Pal *et al*. noted that pain reduction level is more rapid in zinc oxide eugenol group than in PRGF group and saline irrigation group at 1d, 2d, 3d, 7d (*p*<0.001), but the change is no significant at 15d in all the groups. Contrary to the above, King *et al*. indicated that there were not statistically significant for the pain level between PRGF and Alvogyl® group at 3d and 7d, even though the VAS value of the PRGF group was low.

**Table 1:**
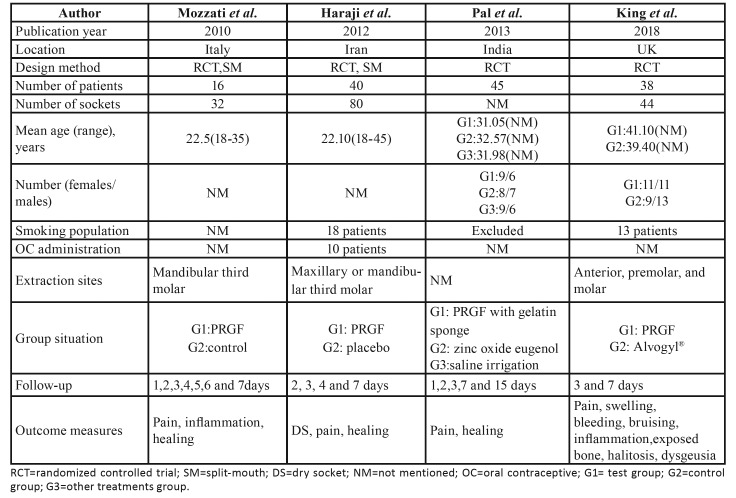
Characteristics of all the selected studies.

**Table 2:**
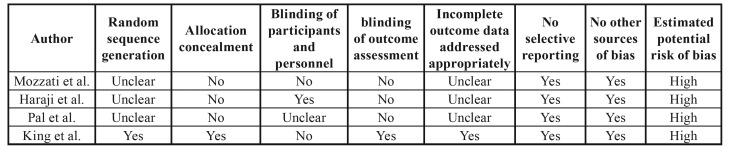
Results of quality assessment.

**Table 3:**
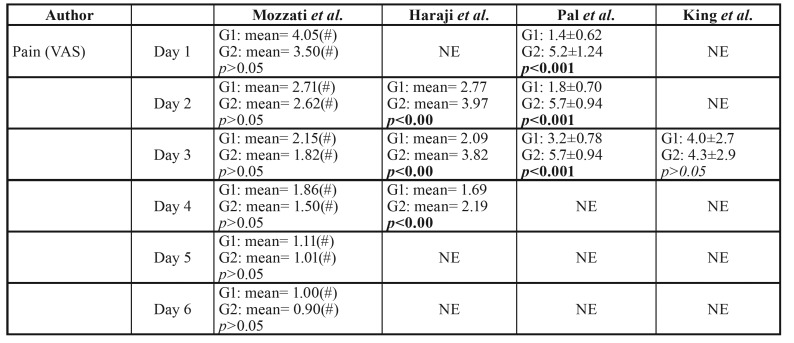
Results of studies included for analysis.

**Table 3 cont:**
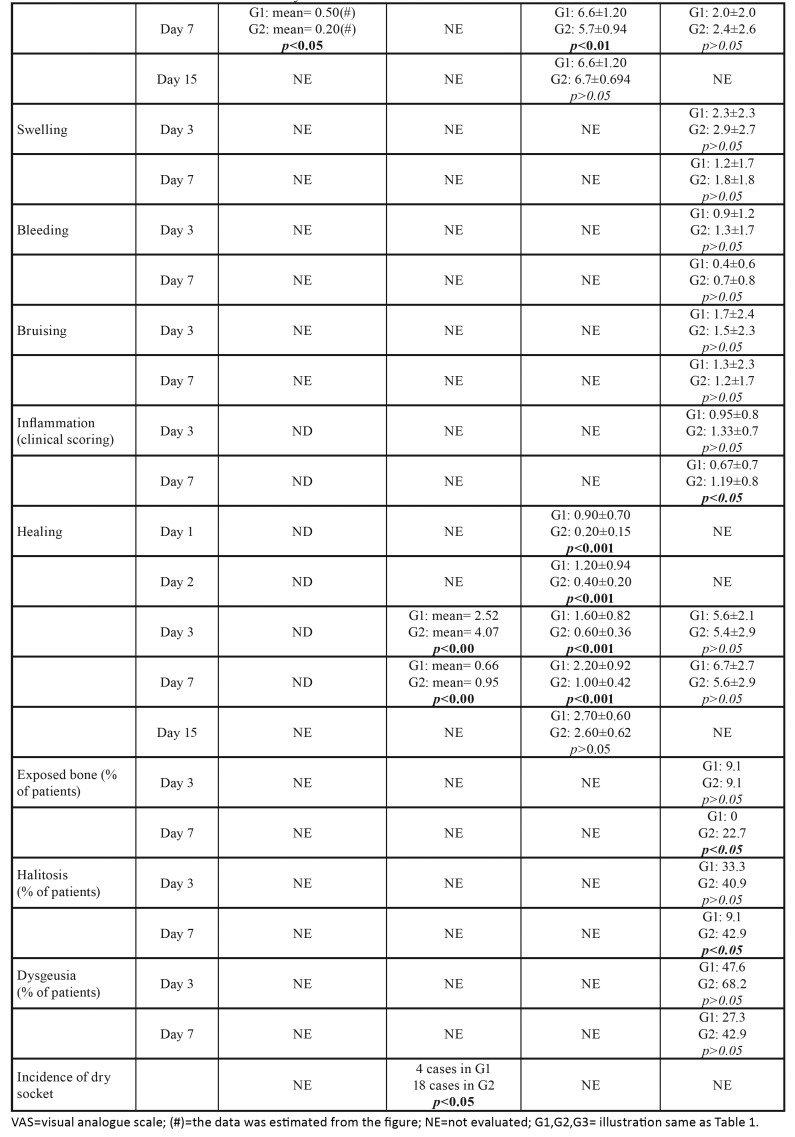
Results of studies included for analysis.

Alveolar fossa healing

During the 7d follow-up periods, Haraji *et al*. and Pal *et al*. found that the healing of the PRGF group was faster than that of the corresponding control group with *p* value <0.001. However, no statistical difference was found in the study by King *et al*. As the postoperative time was prolonged to 15d, the Pal *et al* reported there is no difference in healing values between groups.

Inflammation

King *et al*. indicated that at 7d clinical inflammation was significantly lower in patients in the PRGF® group compared to the Alvogyl® group. Whereas these differences were not significant at 3d.

The incidence of dry socket

Haraji *et al*. evaluated the incidence of dry socket. In the control group, 18 patients presented dry socket. Four patients developed dry socket in PRGF group. This was indicative of a statistically significant difference when PRGF was used (*p*<0.05).

## Discussion

PRGF was used in various types of surgery in oral and maxillofacial surgery. These procedures included series of complicated surgery such as the tooth extraction socket filling (38-40), maxillary sinus augmentation (41), and maxillofacial bone defects (42,43). It has been reported that the use of PRGF can reduce postoperative pain and inflammatory response, accelerate epithelial formation of soft tissue and promote regeneration of bone tissue (44,45).

The purpose of this review is to evaluate the efficacy of PRGF used in the dry socket management following the third molars extraction, further to provide scientific evidence for clinical applications. Although many scholars praised the benefits of PRGF clinical application in many fields, few clinical trials have been conducted to investigate the effect of PRGF on dry socket treatment after tooth extractions, especially randomized controlled trials. Four randomized controlled trials (12,26,34,35) on this topic was retrieved into this review, of which only one article meet the high quality (35) standards of the modified Jadad scale (36) and the remaining three (12,26,34) were low quality. In addition, obvious heterogeneity were found in the method adopted and outcome variables used in the process of evaluation of the healing of dry socket. A quantitative meta-analysis cannot be performed in this case and we could only do a qualitatively descriptive study on the subject.

One possible factor amongst many confounding factors in this systematic review is the choice of indications for extraction. If the teeth were extracted for different reasons such as impact, trauma, periodontitis or periapical infection, the healing process may be different. A histological study (46), conducted for nearly two years, found that the speed of bone formation in the diseased sockets were more slowly than that in the disease-free sockets. New bone area exceeded 50% of the total newly regenerated tissue in the infected sockets after 16 weeks, whereas new bone area in the disease-free sockets exceeded 50% of the total tissue. However, a retrospective chart review by Bell *et al*. (47) demonstrated that clinical attempt to place implant in the extraction socket in the presence of chronic apical periodontitis can be regarded as a safe and feasible treatment option. It is well known that the healing of the impacted third molars with soft tissue resistance would be better than that with bone tissue resistance.

Another confounding factor could be the types of protocol for acquiring PRGF preparations among different studies. Currently, there are many different methods proposed for the preparation of PRGF products. The core aim of different technique used to obtain PRGF was to produce a leukocyte-free preparation so as to reduce the content of pro-inflammatory cytokines. Some of the four included studies did not provide a detailed description of a series of parameters involving in the PRGF production preparation process (cell separators used, centrifugation methods, blood volume collected before surgery, collected the platelet baseline concentration, the amount of platelet concentrate obtained, the final increase in platelet concentration, the type of blood anticoagulant and platelet activator used). Any of these factors may play a role in the vitality and activity of the tissue. Different concentrations and levels of PRGF may have different biological properties.

There are some limitations for this systematic review: 1- the search process led to the inclusion of only four articles, the number of which was too small. In addition, the number of patients included in the study was small, result of which may be partial bias. 2- the included studies did not perform well in random sequences generation, allocation concealment and blinded methods, as these are important for RCTs. Furthermore, there was no multicenter randomized controlled studies. 3- there are no specific diagnostic criteria for dry socket in these studies. 4- oral contraceptive use (48) and tobacco smoking (49) may influence the incidence of dry socket, but he included studies did not handle these two confounding factors.

In summary, meta-analysis cannot be performed given the heterogeneity of the outcome variables included in the different studies. According to results from selected studies, the use of PRGF may help reduce pain and inflammation after tooth extraction, thereby improving the quality of life of patients after tooth extraction. However, after evaluating quality of the included articles, we found that the evidence quality of PRGF applied to the dry socket management after the third tooth extractions was poor, so it was impossible to make a recommendation for clinical use of PRGF. Clearly, a multicenter clinical randomized controlled trial is needed to urgent conduct to evaluate the safety and efficacy of PRGF for dry socket management after mandibular third molar extraction.
